# Rare and extreme outcomes in risky choice

**DOI:** 10.3758/s13423-023-02415-x

**Published:** 2023-11-16

**Authors:** Alice Mason, Elliot A. Ludvig, Marcia L. Spetch, Christopher R. Madan

**Affiliations:** 1 Department of Psychology, University of Bath, Bath, United Kingdom; 2 Department of Psychology, University of Warwick, Coventry, UK; 3 Department of Psychology, University of Alberta, Edmonton, Alberta Canada; 4 School of Psychology, University of Nottingham, Nottingham, UK

**Keywords:** Rare outcomes, Risky choice, Decisions-from-experience, Extreme outcomes, Sampling models

## Abstract

Many real-world decisions involving rare events also involve extreme outcomes. Despite this confluence, decisions-from-experience research has only examined the impact of rarity and extremity in isolation. With rare events, people typically choose as if they underestimate the probability of a rare outcome happening. Separately, people typically overestimate the probability of an extreme outcome happening. Here, for the first time, we examine the confluence of these two biases in decisions-from-experience. In a between-groups behavioural experiment, we examine people’s risk preferences for rare extreme outcomes and for rare non-extreme outcomes. When outcomes are both rare and extreme, people’s risk preferences shift away from traditional risk patterns for rare events: they show reduced underweighting for events that are both rare and extreme. We simulate these results using a small-sample model of decision-making that accounts for both the underweighting of rare events and the overweighting of extreme events. These separable influences on risk preferences suggest that to understand real-world risk for rare events we must also consider the extremity of the outcomes.

## Introduction

What are the odds of being diagnosed with a rare illness, dying in a plane crash, or winning the lottery? All three events are both very unlikely to occur and the outcomes themselves are extreme. To date, decision-making research has generally examined how people respond to event rarity and outcome extremity separately. For example, many studies have examined the effect of rare events on monetary gambles, financial risk-taking ﻿and medical decisions (Lejarraga et al., 2016) as well as on climate action decisions (Liang et al., 2019; Newell et al., 2016; Olschewski et al., 2023). Separate lines of research have examined risk preferences involving extreme outcomes (Konstantinidis et al., 2018; Madan et al., 2014). In the present study, we directly examine how rare and extreme outcomes combine to influence people’s risk preferences when making choices based on past experience.

Typically, in decisions-from-experience tasks, people make repeated choices between a fixed option and a risky option. The risky option leads to one of two possible outcomes, with different probabilities. When the risky option includes rare events (< 20% chance), people consistently choose as if they underweight those rare events (Camilleri & Newell, 2011; Hertwig et al., 2004; Lejarraga & Gonzalez, 2011; Newell & Rakow, 2007; Yechiam & Busemeyer, 2006). This underweighting of the rare event corresponds to the following choice patterns: When the rare event is better than the common event, people choose the safe option more often (i.e., are risk averse), and when the rare event is worse than the common event, people choose the risky option more often (i.e., are risk seeking). Note that this pattern is the opposite of results with decisions from description, where rare events are usually overweighted (e.g., Kahneman & Tversky, 1979).

The underweighting of rare events is thought to be driven by two key processes: a reliance on small samples and recency (Erev et al., 2022). When making decisions from experience, people need to represent and integrate the value of the different options. People typically use small samples of experience to evaluate their options (Hertwig et al., 2004; Plonsky et al., 2015; Wulff et al., 2018), and rare outcomes are less likely to be included in these samples as compared to common outcomes. Models based on this idea of relying on small samples, such as the sample-of-N model, are often used to explain risk preferences with rare outcomes (Erev & Roth, 2014). In these models, people make choices by randomly sampling N outcomes with replacement from all past experiences and then select the option with a higher average outcome. In addition, people give more weight to outcomes they have recently experienced compared to earlier outcomes. One possible explanation for recency in decisions from experience is that people store their experiences in memory and older experiences are likely to decay or suffer more interference (Brown et al., 2007; Hotaling et al., 2022; Oberauer et al. 2016). Such recency effects can further amplify the effects of small samples in producing underweighting of rare events.

The effects of extreme events have also been studied in decisions from experience. An extreme event is defined as the best or worst outcome in the decision context (Ludvig et al., 2014; Madan et al., 2021). For risky choices with extreme outcomes where each outcome is equally likely to occur (*p* =.5), people choose as if they overweight the most extreme (highest and lowest) outcomes in the decision set, drawing them toward risk seeking for higher-valued options and toward risk aversion for lower-valued ones. This decision bias is thought to be driven by a tendency for people to remember the highest and the lowest outcomes in the set (Madan et al., 2014, 2021). People show this tendency to overweight the most extreme outcomes in both risky-choice tasks and non-preferential tasks (Mason et al., 2022), suggesting that this decision bias reflects a basic cognitive process to overweight extreme outcomes in memory (Lichtenstein et al., 1978).

In decisions from experience, people need to form mental representations of their environment and integrate the value and frequency of different options. Many theories and formal models of risky choice assume that people’s preferences for different options are formed by sampling from their past experiences. In most cases, people are assumed to only maintain a small sample of past events and the outcomes that are included in this sample will ultimately guide their preferences and choices (Erev et al., 2022). With rare events, the assumption is that the rare events will be under-represented in the sample and therefore underweighted. In contrast, extreme events are more likely to be remembered and to be included in a sample (Madan et al., 2014), which could be an optimal way of allocating limited resources by prioritising the most important eventualities (Bhui et al., 2021; Lieder & Griffiths, 2018; Vanunu et al., 2021).

Past research has examined how people make decisions from experience regarding both rare and extreme outcomes, but not how these two decision biases combine. To examine the impact of both rare and extreme outcomes on risky choices, we designed a set of options that varied the extremity of the rare event (i.e., whether the rare event was also the lowest or highest number in the decision set). Consider the options shown in Table 1. For the rare non-extreme group, the rare outcomes (in bold) are distinct from the extreme outcomes [-40, -36, **0**, +36, +40]. For the Rare-Extreme group, the rare outcomes are also the most extreme [**-40**, -4, 0, **+**4, **+40**]. For both groups, people should behave as if they are underweighting the rare outcomes. For the Rare-Non-Extreme group, this underweighting would lead to risk seeking for gains and risk aversion for losses. For the Rare-Extreme group, this underweighting would lead to the opposite pattern: risk aversion for gains and risk seeking for losses. The small-sample models of decisions from experience predict the underweighting of rare events, but are not able to account for the overweighting of extreme events. For the design outlined above, these models would predict overweighting of rare events across all conditions, which would produce an interaction in risk preferences between the two groups (rare extreme and rare non-extreme) across the gains and losses.

**Table 1:** Decision problems used in experiment. Participants were assigned to either the Rare-Extreme or the Rare-Non-Extreme group. Both groups were presented with gain and loss choices. The fixed options were always equal to the expected values of the risky options. Rare outcomes occurred 10% of the time. The extreme outcomes (in bold) were +40 and -40, and the non-extreme outcomes were 0

Group	Options	Gains	Gains results	Losses	Losses results
Rare-Extreme	Fixed Risky	+4 90% 0, 10% **+40** [Rare = Better]	*Risk Averse*	-4 90% 0, 10% **-40** [Rare = Worse]	*Risk Seeking*
Rare-Non-Extreme	Fixed Risky	+36 90% **+40,** 10% 0 [Rare = Worse]	*Risk Seeking*	-36 90% **-40**, 10% 0 [Rare = Better]	*Risk Averse*

If, however, people also overweight extreme events, then there should be an additive effect of underweighting of rare events and overweighting of extreme events. For the Rare-Extreme group, this underweighting should be reduced when the rare events are also the extremes, due to the *overweighting* of the extreme outcomes (e.g., Ludvig et al., 2014). On this basis, people in the Rare-Non-Extreme group should be even more risk seeking for gains compared to losses (due to the underweighting of rare 0 events). In contrast, in the Rare-Extreme group, the underweighting of rare events (+/- 40 events) would lead people to be risk averse for gains and risk seeking for losses, but these patterns should be attenuated if people also overweight the extreme events (+/- 40 events).

## Methods

### Participants

A total of 250 participants (183 women; age: M = 19.4 years, SD = 2.5 years) were recruited from the University of Alberta psychology participant pool. Informed consent was obtained, and participants received partial course credit and a cash bonus for participating. Participants were instructed in groups of up to 15, but performed the task in individual rooms. Procedures were approved by the University of Alberta Research Ethics Board. This sample size is based on effect sizes in previous work and gives 95% power to find an effect size *d* = 0.46 with alpha = 0.05 in a between-groups design.

### Procedure

**Fig. 1:**
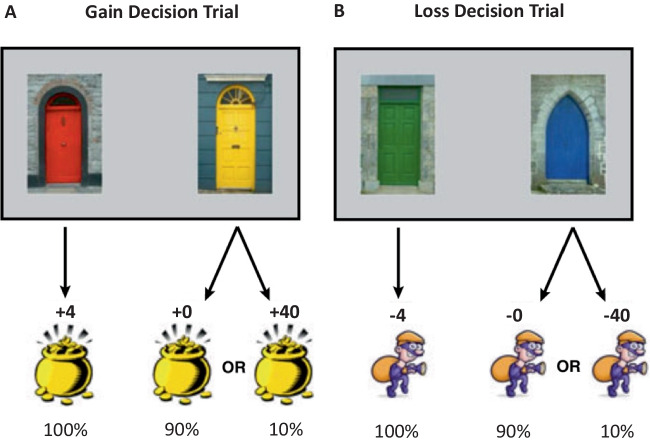
Schematic representation of the general method used. Specific values correspond to the Rare-Extreme Group. Decision trials involved choices between two gain doors (**A**) or two loss doors (**B**). One door always led to a gain (or loss) of a fixed number of points, and the other door led to one of two possible outcomes with the probabilities indicated (90% or 10%). Choices were followed by feedback about the amount gained or lost from the selected door and counterfactual feedback about the non-selected door

All testing was performed using Windows PCs running E-Prime. Participants played a computer-based task to earn points that were exchanged for money. On each trial, participants were presented with pictures of one or two visually distinct doors, which they clicked on to obtain an outcome. Clicking a door was immediately followed by removal of the door images and 1.2 s of feedback showing the number of points won or lost (see Fig. 1). The experiment consisted of six blocks of trials, which were separated by a brief break (an on-screen riddle).

The task used a full-feedback procedure in which all choices were consequential, and feedback for both options was provided. The total accumulated points were continuously displayed at the bottom of the screen. Trials were separated by either a 1-s or a 2-s interval.

There were four choice options: a fixed gain, a risky gain, a fixed loss, and a risky loss. Participants were randomly assigned to one of two experimental groups: Rare-Non-Extreme or Rare-Extreme. In the Rare-Non-Extreme group, for gains, the risky option was a 90% chance of +40 and a 10% chance of 0. Thus, the rare outcome was the worse of these two gains. The fixed option had the same expected value as the risky option and always provided +36 points. For losses, there was a 90% chance of -40 and a 10% chance of 0, meaning that the rare outcome was the better of these two losses. The fixed option always yielded -36 points. In the Rare-Extreme group, for gains, the risky option was 90% chance of 0 or a 10% chance of +40 [Rare = Better], and the fixed option was +4. For losses, the risky option was 90% chance of 0 or a 10% chance of -40 [Rare = Worse], and the fixed option was -4. The outcomes associated with each door were counterbalanced across participants, and the left-right location of each outcome was counterbalanced for each trial type. The order of trials varied randomly within each block.

There were six blocks of trials each consisting of 48 *decision* trials and 12 *catch* trials. The *decision* trials provided a choice between a risky option and a fixed option of equal expected value (i.e., both options were gains or both options were losses). The *catch* trials required a choice between options with substantially different expected values (e.g., risky gain vs. fixed loss). These trials provided a manipulation check that participants had learned the contingencies and were choosing to maximize points/money. As per our standard practice, all participants who picked the reward-maximizing option on fewer than 60% of the catch trials were excluded from all results (e.g., Ludvig & Spetch, 2011; Madan et al., 2019). Data from nine of the 250 participants were excluded from the analysis because these participants scored less than 60% in the catch trials. The primary dependent variable was the proportion of risky choices in the final three blocks, after participants had had the chance to experience and learn the relevant contingencies.

## Results

**Fig. 2:**
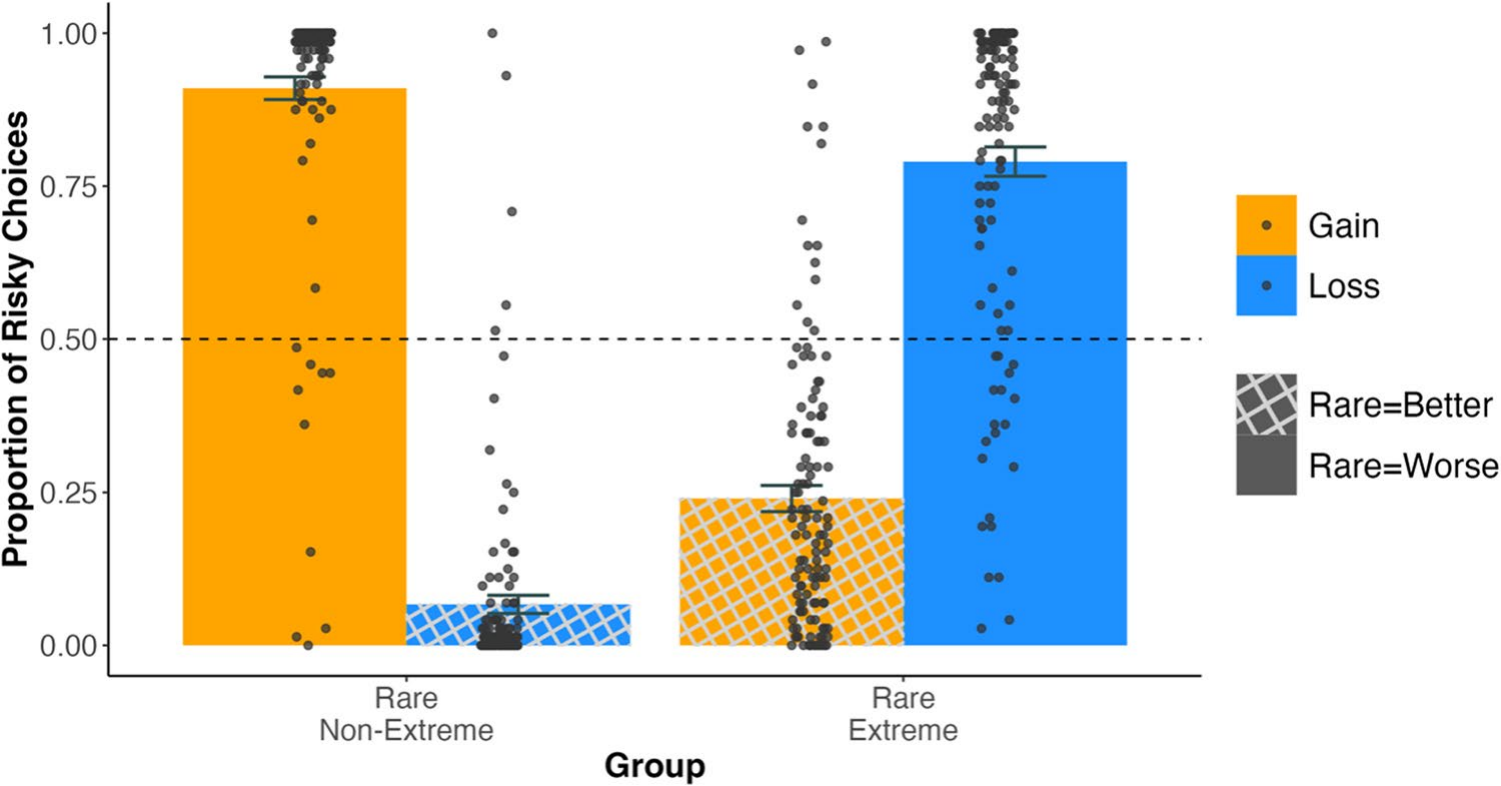
Proportion of risky choices made by the Rare-Non-Extreme and Rare-Extreme groups for the gain and loss trials across the last three blocks. The error bars indicate the standard errors of the mean. The Rare-Non-Extreme group was more risk seeking for gains than losses. In contrast, the Rare-Extreme group was more risk seeking for losses than gains. The hashed pattern indicates conditions where the rare outcome was better than the fixed outcome. The dashed line indicates risk neutrality. Each grey dot represents an individual participant

Figure 2 plots the proportion of times people in the Rare-Non-Extreme and Rare-Extreme groups selected the risky option for gains and losses. The results are consistent with the underweighting of rare events, whereby people pay less heed to the event that happens less often. For the Rare-Non-Extreme group, this underweighting means behaving as if they are ignoring the rare (and worse) 0 outcome and showing risk seeking for gains [Rare = Worse], selecting the risky option 91.3 ± 2.0% (Mean ± Standard Error) of the time. For losses [Rare = Better], when the 0 outcome was rare, people were risk averse and selected the risky option only 6.7 ± 2.0% of the time, again acting as though they underweighted the rare, 0 outcome. For the Rare-Extreme group, the rare outcome was +/- 40. The pattern observed for the Rare-Extreme group was also consistent with underweighting of rare events and the idea that people ignore the big win or loss. In line with this underweighting, the Rare- Extreme group exhibited the opposite pattern: Participants were risk averse for gains [when Rare = Better] and chose the risky option 24.4 ± 2.0% of the time, but were risk seeking for losses [when Rare = Worse], choosing the risky option 79.1 ± 2.0% of the time.

A 2 × 2 (Group [rare non-extreme, rare extreme] × Domain [gain, loss]) between-groups ANOVA showed a significant interaction between domain and group (*F*(1, 239) = 926.40, *p* < .001, *η*_*p*_^*2*^ = .80) and a main effect of domain (*F*(1, 239) = 45.54, *p* < .001, *η*_*p*_^*2*^ = .15), but no reliable effect of group (*F*(1, 239) = 2.88, *p* = .09, *η*_*p*_^*2*^ = .015). Follow-up pairwise *t-*tests comparisons for the main effect of domain and the interaction were all significant (see Table 2 for details).

**Table 2:** The results of the follow-up Welch’s *t*-tests for the main effect of domain and the interaction between domain and group

Condition 1	Condition 2	*t*-statistic	df	*p*	Cohen’s *d*
Rare-Non-Extreme Gain [Worse]	Rare-Extreme Gain [Better]	23.7	232.8	<.001	3.05
Rare-Non-Extreme Loss [Better]	Rare-Extreme Loss [Worse]	-25.7	195.7	<.001	3.34
Rare-Non-Extreme Gain [Worse]	Rare-Non-Extreme Loss [Better]	35.7	232.5	<.001	2.72
Rare-Non-Extreme Gain [Worse]	Rare-Extreme Loss [Worse]	4.0	222.4	<.001	0.52
Rare-Extreme Gain [Better]	Rare-Non-Extreme Loss [Better]	6.8	209.6	<.001	0.88
Rare-Extreme Gain [Better]	Rare-Extreme Loss [Worse]	-17.0	231.0	<.001	1.38

For each condition the words in square brackets indicate whether the rare outcome in the risky option is “[Better]” or “[Worse]” than the fixed option

Note how when the rare outcomes were also the extreme outcomes, the underweighting was reduced. This reduction can be seen by specifically comparing the conditions where people were either risk seeking or risk averse. In both the losses condition in the Rare-Extreme group [Rare = Worse] and the gain conditions for the Rare-Non-Extreme group [Rare = Worse], people were risk seeking in line with underweighting. In the Rare-Extreme losses condition, however, people were 12.2 ± 2.8% less risk seeking than in the Rare-Non-Extreme gains condition (*t*(238) = -4.34, *p* <.001, *d* = 0.56). Similarly, in both the Rare-Extreme gains condition [Rare = Better] and the Rare-Non-Extreme losses [Rare = Better], people were risk averse, in line with underweighting of rare events. In the Rare-Extreme gains condition [Rare = Better], where the rare event was also extreme, this risk aversion was attenuated and people were 17.7% ± 2.8% more risk seeking (*t*(239)= 6.30, *p* <.001, *d* = 0.82).

## Simulation

**Fig. 3:**
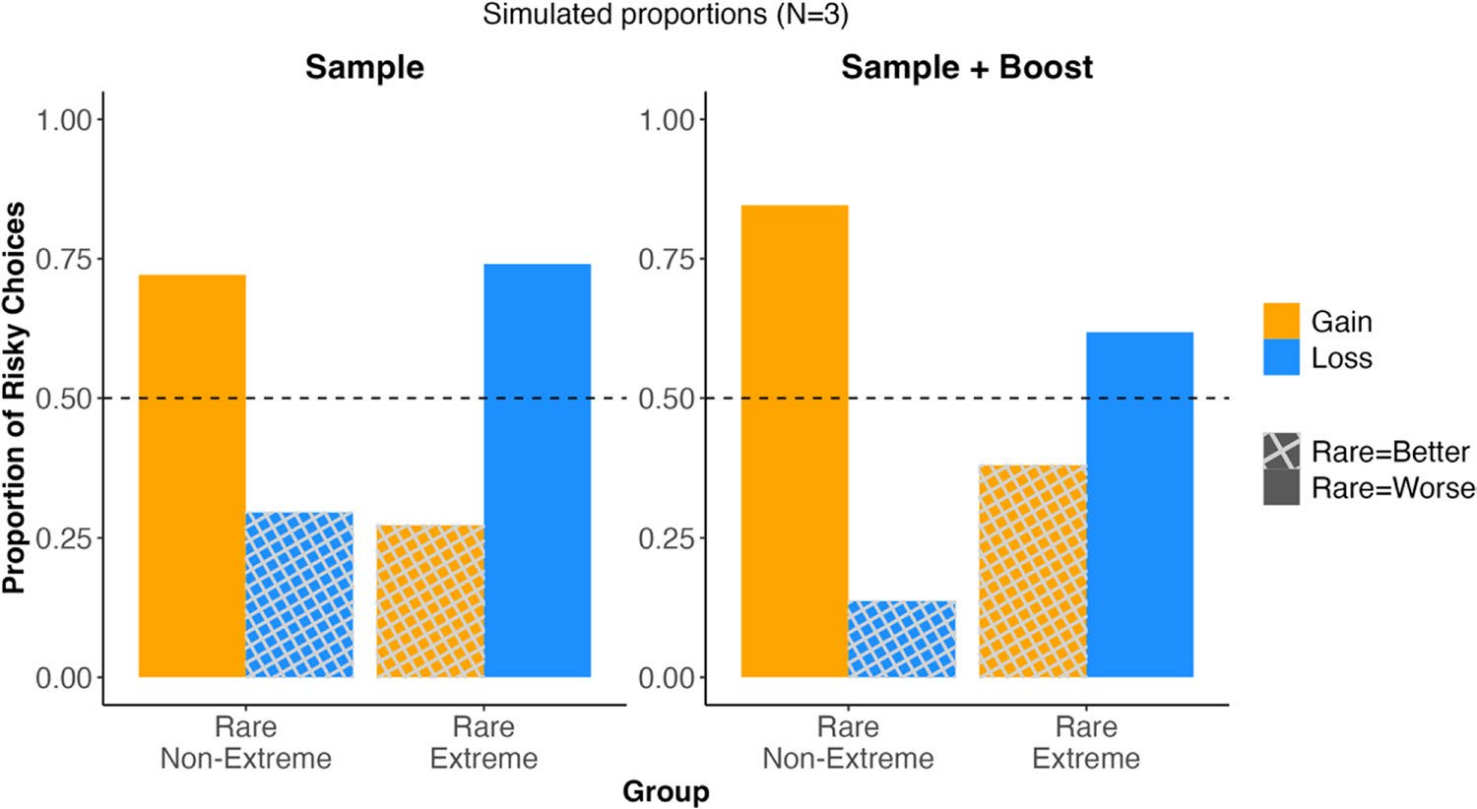
Simulated proportion of risky choices according to two sampling models. The first two pairs of bars show the predictions of the “Sample” model with a sample size of 3 for the choices in the Rare-Non-Extreme and the Rare-Extreme groups. The second pair of bars shows the predictions for the “Sample+Boost”, which also uses a sample of 3, but increases the probability of sampling the extreme items. The hashed pattern indicates conditions where the rare outcome was better than the fixed outcome. A full set of simulations for models with sample sizes from 1 to 5 are shown in the Appendix Fig. 4

For both the rare-non-extreme and rare-extreme groups, we simulated risk preferences using a Reliance-on-small-samples model: the sample-of-N model (Erev & Roth, 2014). Figure 3 shows the model-predicted pattern of risk preferences for these groups using a sample size of 3, which provided the best account of the data (see Appendix Fig. 4, which shows the simulations for sample sizes of 1, 2 ,4 and 5). In these models, the rare outcomes have a lower probability of being included in the small sample. As a result, the rare outcomes are underweighted and for the Rare Non-Extreme group, there is more risk seeking for gains and risk aversion for losses. This pattern is reversed in the Rare Non-Extreme group, where there is risk aversion for gains and risk seeking for losses.

This pattern qualitatively matches human behaviour (see Fig. 2), but this sample-only model is not sensitive to the extremity of the rare outcomes, diverging from what people do. To include this sensitivity to extremes, we also simulated a model that increases the probability that the extreme outcomes are included in the sample of 3. In this simulation, the probability of including extreme outcomes in the sample was “boosted” by 5%. The exact percentage used only quantitatively changes the predictions, but the pattern is consistent. As can be seen in the right panel of Fig. 3, this model underweights the rare event less, amplifying the difference in risk preference for gains and losses in the Rare-Non-Extreme group, but moderating it in the Rare-Extreme group. This pattern better matches the results from humans who show a similar change in risk preferences.

## Discussion

This experiment demonstrates how two known biases in decision from experience – the underweighting of rare events (Hertwig & Erev, 2009) and the overweighting of extreme outcomes (Ludvig et al., 2014) – combine in risky choice. The two biases were additive, but the effect of rare events was considerably larger. Thus, in decision problems involving rare outcomes, as expected, people behaved as though they underweighted the rare events: they chose as though the rare event happened less often than it actually did. When the rare event was also an extreme outcome, however, this underweighting effect was attenuated as compared to when the rare event was not an extreme outcome.

Underweighting of rare outcomes in decisions from experience is a well-established phenomenon (Erev et al., 2022; Hertwig & Erev, 2009). Here we extend these results using a new protocol where visual cues predict outcomes and gain and loss problems are intermingled. This study systemically varied the decision problems used to examine the influence of both rare and extreme events (see Table 1). Whilst the results indicate that both decision biases were present, the effects of rare outcomes were stronger. A potential explanation for these decision biases is that people use small, biased samples to inform their decisions. In the case of rare outcomes, people seem to rely on small samples, which may not include the rare but consequential events (Pleskac & Hertwig, 2014; Taleb, 2010). This mechanism is also evident in models of decisions from experience that assume that the value of an option is determined by a subset of samples (Erev et al., 2022; Plonsky et al., 2015; Wulff & Pachur, 2016). In some models, these samples are randomly selected and in others the most recent outcomes are used. In both types of model, however, there is a lower probability that the rare event will be included in the sample.

The patterns observed in experience-based choice tasks systematically diverge from the risk preferences when the odds and outcomes of the gambles are described (Kahneman & Tversky, 1979). For described gambles with rare outcomes, people instead overweight the rare outcome. One potential explanation for this difference is that in experienced-based choices people must rely more on learning and memory (Rakow & Newell, 2010). The recency mechanism adopted in many models of decisions-from-experience and the notion that people are likely to sample more recent events is compatible with memory-based mechanisms including recency, decay and retroactive interference (Brown et al., 2007; Farrell, 2012; Hotaling et al., 2022). Similarly, the overweighting of extreme outcomes in choice is thought to reflect a memory bias to overweight the most extreme outcomes (Madan et al., 2014, 2021; Madan, 2024).

Our findings highlight that models that assume that sampling from past experience is random (e.g., Erev et al., 2022) do not wholly account for risk preference when outcomes are also extreme. In existing models, by virtue of having occurred less often, such extreme events would not be included in random or recent samples. Instead, the sampling process needs to be adjusted to actively favour outcomes at the ends of the distribution – for example using utility-weighted sampling (Lieder et al., 2018; Vanunu et al., 2021) or a memory priming mechanism as used in the MEM-EX model, which suggests that people may preferentially sample such salient items from memory (Hotaling et al., 2022). Our previous work looking at extreme outcomes suggests that the most extreme items and their neighbours would be preferentially sampled (Ludvig et al., 2018).

Our simulation (see Fig. 3) demonstrates that a model that includes both underweighting of rare outcomes and overweighting of extreme outcomes (Sample + Boost) is better able to account for the behavioural patterns. We have hypothesised that this overweighting of extreme outcomes is driven by a gist-based memory bias toward those outcomes (Ludvig et al., 2018; Mason et al., 2022). Such a gist-based process implies that the samples themselves may not be veridical, providing a further angle for future sample-based models.

As in the present results, previous work has firmly established that people choose as if rare outcomes are underweighted in decisions from experience (Hertwig et al., 2004; Hertwig & Erev, 2009). As a result, people are typically risk averse for rare gains because they act as if they ignore the big win, and risk seeking for rare losses because they act as if they ignore the big loss. Despite this underweighting, there are many everyday decision scenarios where people do include rare events in their small sample. People choose to gamble on the off-chance they will win, and choose not to swim in the sea for fear of sharks, despite perhaps having plenty of experience with the rarity of these events. Our work demonstrates that the extremity of a rare event can shift people away from the traditional risk patterns for rare events in experience. Understanding how rare and extreme events together shape risk preferences brings us closer to understanding risk preferences in these real-world scenarios.

## Figures and Tables

**Fig. 4:**
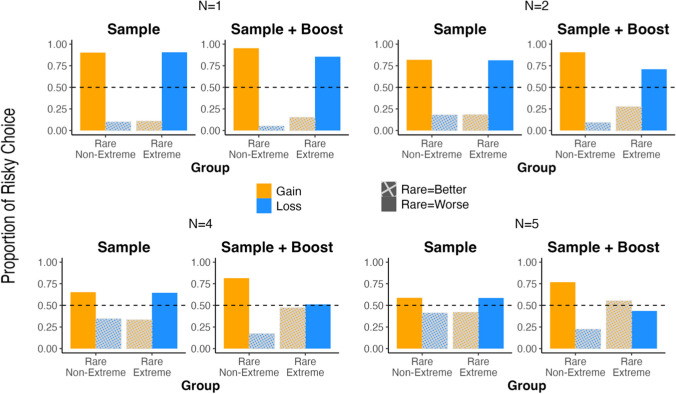
Simulated proportion of risky choices according to two sampling models using sample sizes 1, 2, 4 and 5 (see Fig. 3 in main text for sample size 3). For each plot, the first two pairs of bars show the predictions of the corresponding “Sample” model for choices in the Rare-Non-Extreme and the Rare-Extreme groups. The hashed pattern indicates when the rare outcome is better than the safe outcome. The second pair of bars shows the predictions for the “Sample” model that also uses N samples, but increases the probability of sampling the extreme items
